# Comparison of Mechanical and Corrosion Properties Between Coarse-Grained and Ultrafine-Grained High-Strength Aluminum Alloys

**DOI:** 10.3390/ma19020407

**Published:** 2026-01-20

**Authors:** Xiaolian Zhao, Yiwen Shao, Guoxiang Xu, Tong Liu, Dong Liu, Guoqiang Lin

**Affiliations:** 1 School of Resources, Environment and Materials, Guangxi University, Nanning 530004, China; 2 Key Laboratory of High Performance Structural Materials and Thermo-Surface Processing, Education Department of Guangxi Zhuang Autonomous Region, Guangxi University, Nanning 530004, China

**Keywords:** high-strength aluminum alloys, strength, elongation, corrosion

## Abstract

**Highlights:**

**What are the main findings?**

- A 7A52 aluminum alloy was processed by multi-axial forging and aging.
- The fine-grained alloy shows higher strength, elongation and corrosion resistance.
- The strengthening is due to dislocation multiplication and grain size reduction.
- The improvement in grain boundary precipitates enhances corrosion resistance.

**What are the implications of the main findings?**

- This study provides a feasible strategy to improve the comprehensive properties of high-strength aluminum alloys
- The severe plastic deformation and heat treatment optimizes the microstructure of alloys, thus enhances their overall performance.
- Multi-axial forging and aging treatment features excellent engineering applicability and can be extended to industrial production of high-performance aluminum alloy components.

**Abstract:**

Multi-axial forging (MAF) and aging were employed to process a high-strength aluminum alloy. The tensile properties, microstructure, and corrosion behavior were researched. After MAF, the strength of the alloy was observably increased, but the elongation was decreased. The strengthening mechanism resulted from dislocation multiplication and grain size reduction. After aging, strength was enhanced further, and elongation was improved. The strength and elongation are 561 MPa and 12.3%. Moreover, the corrosion resistance was obviously enhanced. The further strengthening is mainly attributed to the precipitation strengthening. The larger size and discontinuous distribution of grain boundary precipitates resulted in the alloy having higher corrosion resistance.

## 1. Introduction

In aerospace and military industries, high-strength aluminum alloys have been extensively used because of their superior specific strength and excellent weldability [1,2]. Nevertheless, these alloys are prone to different forms of localized corrosion, including pitting corrosion, intergranular corrosion (IGC), stress corrosion cracking (SCC), and exfoliation corrosion (EXCO). Therefore, the significance of improving the corrosion resistance of high-strength aluminum alloys cannot be overemphasized. In fact, the corrosion resistance can be significantly improved through the over-aging, retrogression, re-aging, and thermo-mechanical treatments at the expense of sacrificing strength or elongation [3,4,5]. Therefore, it becomes interesting to achieve high strength, elongation, and corrosion resistance of the high-strength aluminum alloys.

The size and distribution of the precipitates in the 7050, 7150, and 7075 aluminum alloys have an important influence on the tensile properties and corrosion resistance [6,7]. Moreover, ultrafine-grained (UFG) materials fabricated by methods of severe plastic deformation (SPD) show unique physical, chemical, and mechanical properties [8,9]. Recently, some scholars have utilized the severe plastic deformation technique to process 5083 aluminum alloy, resulting in an increase in its strength and corrosion resistance [10]. In general, the materials processed by SPD show lower elongation [11,12,13]. Plastic deformation may often cause microstructural refinement and dislocation multiplication in high stacking-fault energy metals. SPD and heat treatment may improve the microstructure of high-strength aluminum alloys, for instance, in terms of the size and interparticle distance of precipitates within the grain and on the grain boundary. It will be significant that the high-strength aluminum alloys exhibit higher strength, elongation, and corrosion resistance produced by means of SPD and thermal treatment.

The multi-axial forging (MAF) processing is more suitable for engineering applications. The 7A52 aluminum alloy, with its outstanding mechanical properties, corrosion resistance, and lightweight characteristics, has become a key structural material in the aerospace, rail transportation, and high-end equipment manufacturing. In this work, the multi-axial forging (MAF) and aging were used to process 7A52 aluminum alloy. The microstructure, hardness, tensile, and corrosion properties were researched. On this basis, the hardness, tensile, and corrosion properties between the CG sample and the UFG sample were compared and discussed. It is expected that there will be a strategy to improve the comprehensive properties of high-strength aluminum alloys.

## 2. Materials and Methods

The alloy tested in the experiments was a commercial 7A52 aluminum alloy rod (Guangxi Nannan Aluminum Processing Co., Ltd., Nanning, China), and the chemical composition is listed in Table 1. The CG samples were heated at 470 °C for 2 h and quenched in water, and then heated at 120 °C for 24 h (T6-processed). Forging blanks were machined into squares of a size of 20 mm × 20 mm × 20 mm and were then heated at 470 °C for 2 h and quenched in water. They were then preheated at 150 °C for 20 min, 10 min, and 10 min before the samples were forged in the first, second, and third passes. Alternate forging was carried out in three mutually perpendicular directions (x-y-z-x…) of the sample, and a total of three passes were forged. MAF was conducted at a strain rate of approximately 10 s^−1^. About 17% compression was produced in every pass of forging. MAF was performed by a hydraulic press machine. To simultaneously achieve higher strength and elongation, the samples were heated at 120 °C for 22 h after forging.

The hardness was tested using an AVH-5L microhardness tester (Shanghai Hengyi Precision Instruments Co., Ltd., Shanghai, China) by employing a 0.29 N load for 15 s. Tensile properties were tested via a materials testing machine (Shenzhen SANS Testing Machine Co., Ltd., Shenzhen, China) operating at a constant rate of 1 mm/min. At least three reproducible tests were conducted for each type of sample. Dog-bone-shaped tensile specimens of 5 mm in gauge length, 1 mm in width, and 1 mm in thickness were cut. On the basis of the Chinese Standard GB/T 7998-2005 [14], IGC susceptibility was evaluated by using a solution. The solution was prepared using 57 g of NaCl, 10 mL of H_2_O_2_, and 1 L of distilled water. The samples were maintained in the solution for 6 h at 35 °C. On the basis of the Chinese Standard GB/T 22639-2008 [15], the EXCO property was compounded by using a solution. The corrosion solution was NaCl 4.0 mol/L, KNO_3_ 0.5 mol/L, and HNO_3_ 0.1 mol/L, and the residual was distilled water. The samples were maintained in the solution for 48 h at 25 °C. At least three reproducible corrosion tests were conducted for each type of sample.

The polarization curves were measured on a standard three-electrode CHI660E electrochemical workstation (Shanghai Chenhua Instrument Co., Ltd., Shanghai, China). The reference and auxiliary electrodes were a saturated calomel electrode and a platinum sheet; the exposed area for the test was 1 cm^2^, and the corrosion solution was a 3.5 wt% NaCl solution. The electrolyte used in the experiment had a volume of 350 mL. To ensure that the electrochemical system was in a stable state, the specimens were maintained in the electrolyte at the open-circuit potential stable state for 30 min before measuring the polarization curves. When measuring the polarization curve, the scanning rate during the experimental test was 1 mV/s, and the scanning range was set at ±0.3 V of the stable open-circuit voltage value. Five reproducible parallel tests were performed for each type of sample to ensure the reliability of the potential polarization test results.

XRD texts were performed on an X-ray diffractometer (Rigaku Co., Ltd., Tokyo, Japan) with a Cu target. The corrosion morphology of the samples was observed by scanning electron microscope (SEM) (Hitachi, Ltd., Tokyo, Japan). The microstructures of the samples were examined by transmission electron microscope (TEM) (Thermo Fisher Scientific, Waltham, MA, USA).

## 3. Results and Discussion

**Figure 1:**
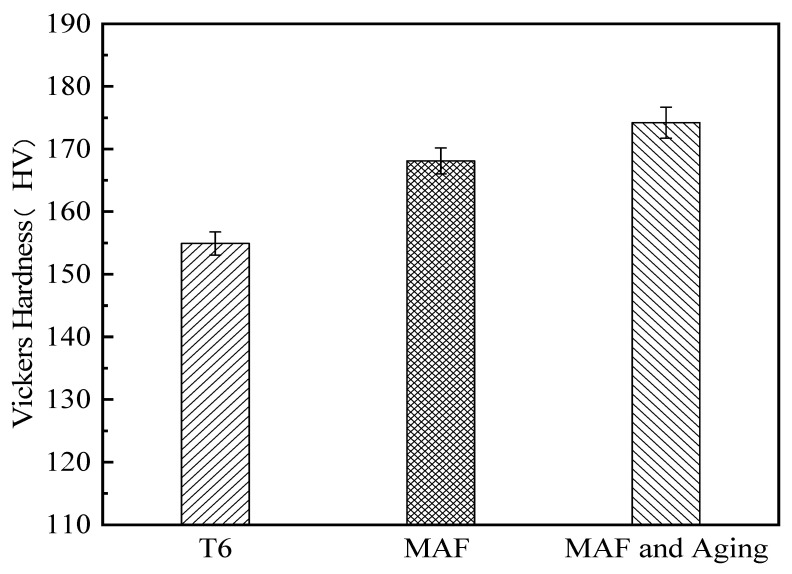
Vickers’ hardness of samples.

**Figure 2:**
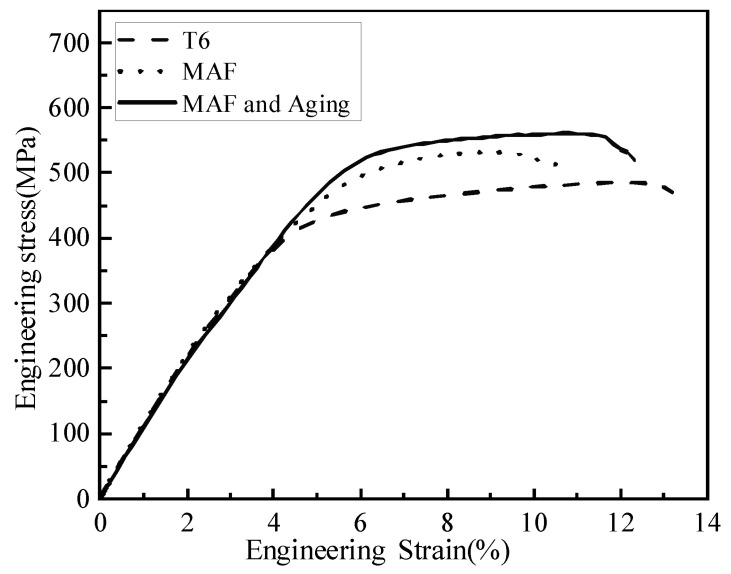
Engineering stress–strain curves of samples.

Figure 1 presents the hardness of the samples. The hardnesses of T6-processed and MAF and aging samples are 154 HV, 168 HV, and 174 HV, respectively. Figure 2 presents the tensile test results of the samples. The strength and elongation of the T6-processed sample reached 486 MPa and 13.2%, respectively. Obviously, the sample presents disappointingly low strength but high elongation. However, the strength was markedly increased, and elongation was greatly reduced after MAF processing. The strength and elongation of the MAF sample are 532 MPa and 10.6%, respectively. The change in strength and elongation is consistent with that of UFG materials fabricated by other SPD ways [16,17]. The strength and elongation of the MAF and aging sample are 561 MPa and 12.3%, respectively. Compared to the T6-processed sample, the strength of the MAF and aging sample was observably increased, but the elongation was slightly decreased.

**Figure 3:**
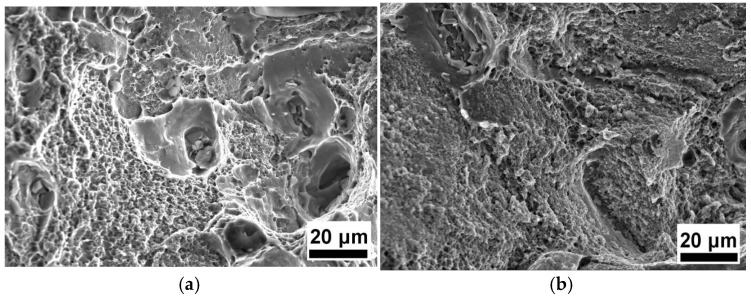
Fractograph of the tensile samples of (**a**) T6-processed and (**b**) MAF and aging specimens.

Figure 3 shows a fractograph of the tensile samples. The fracture features of the dimple and intergranular fracture were found in the T6-processed sample, as presented in Figure 3a. The fracture characteristics of the dimple and intergranular fracture were found in the MAF and aging sample, which are similar to those of the T6-processed sample, as shown in Figure 3b. In addition, the size of the dimple is larger in the fracture of the MAF and aging sample.

**Figure 4:**
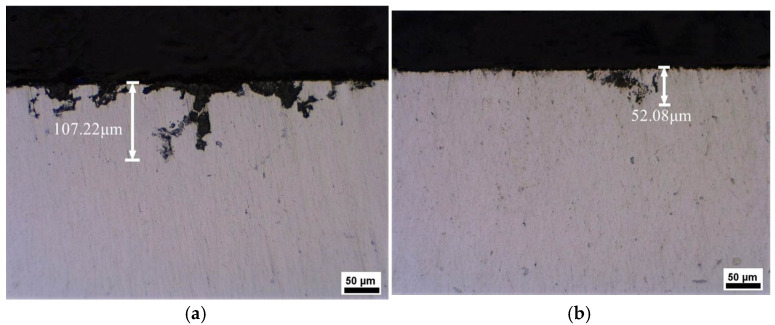
Cross-sectional corrosion profiles of samples after 6 h of immersion in IGC solution: (**a**) T6-processed; (**b**) MAF and aging.

**Figure 5:**
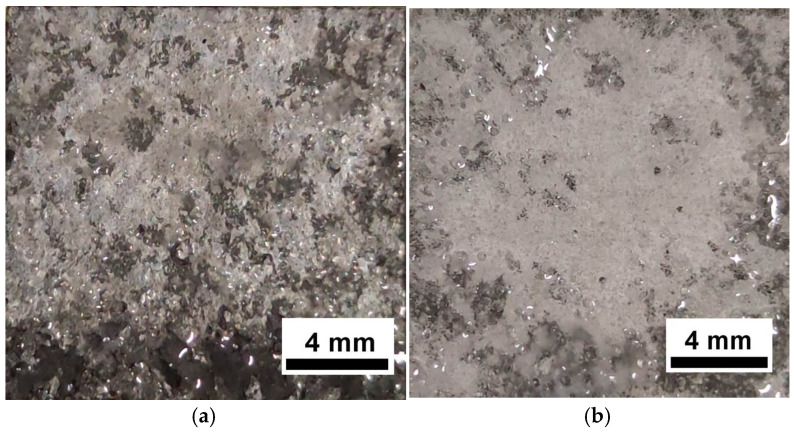
Corrosion morphology of samples after 48 h of immersion in EXCO solution: (**a**) T6-processed; (**b**) MAF and aging.

**Figure 6:**
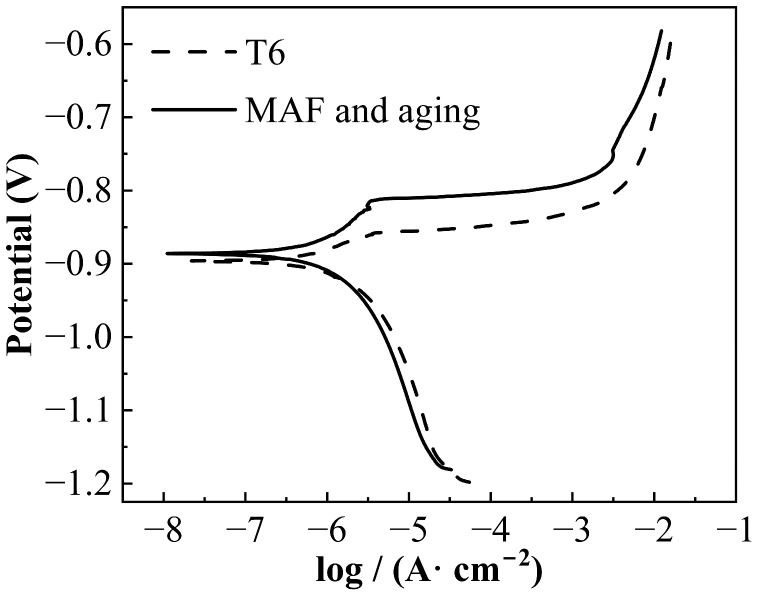
Polarization curves of samples.

**Figure 7:**
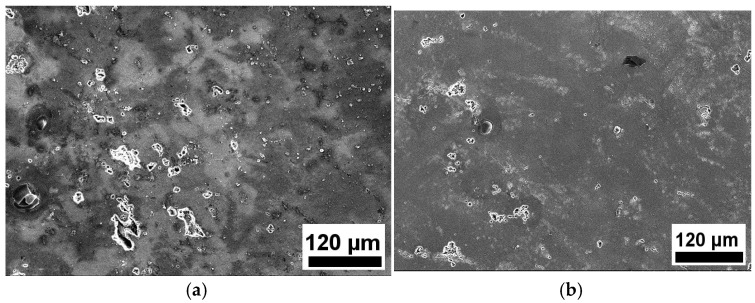
Surface morphology of samples after electrochemical corrosion: (**a**) T6-processed; (**b**) MAF and aging.

Figure 4 presents the IGC morphology of the samples. The depths of corrosion of the T6-processed sample and MAF and aging sample are 107.22 μm and 52.08 μm, respectively. Figure 5 illustrates the exfoliation corrosion profiles of the samples after 48 h of immersion in an EXCO solution. Corrosion pits exist on the EXCO surface of the samples. Compared with the T6-processed sample, there are fewer corrosion pits on the surface of the MAF and aging sample. Obviously, MAF and aging significantly enhanced the IGC and EXCO resistance of the alloy. Figure 6 presents the polarization curves of the samples in a 3.5% NaCl solution. There are differences between the two polarization curves. For the T6-processed sample, the corrosion potential (E_corr_) is relatively higher. Obviously, it can be observed that there are passivation zones. Table 2 shows the electrochemical parameters calculated from the test data. E_corr_, I_corr_, and R_corr_ are corrosion potential, corrosion current density, and polarization resistance, respectively. In fact, the lower the corrosion potential, the higher the corrosion current density, the lower the polarization resistance, the greater the corrosion tendency, the faster the corrosion rate, and the lower the corrosion resistance [18,19,20,21]. Obviously, the MAF and aging sample shows higher corrosion resistance. Figure 7 shows the surface morphology of the sample after electrochemical corrosion. Compared with the T6-processed sample, there are smaller and fewer corrosion pits on the surface of the MAF and aging sample.

**Figure 8:**
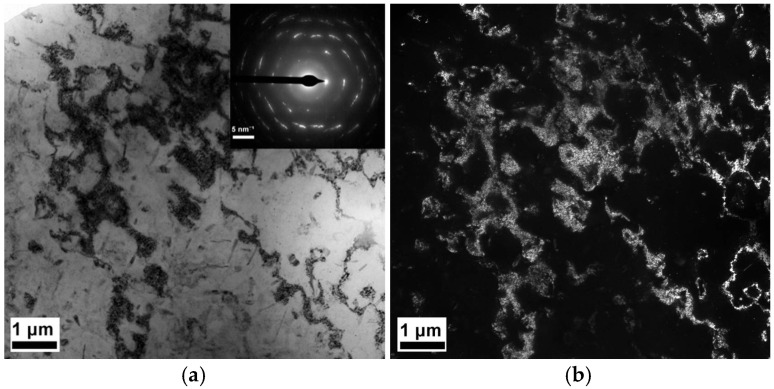
Selected electron diffraction patterns of intracrystalline dislocation distribution and polycrystalline organization of MAF and aging sample: (**a**) bright-field image and (**b**) dark-field image.

**Figure 9:**
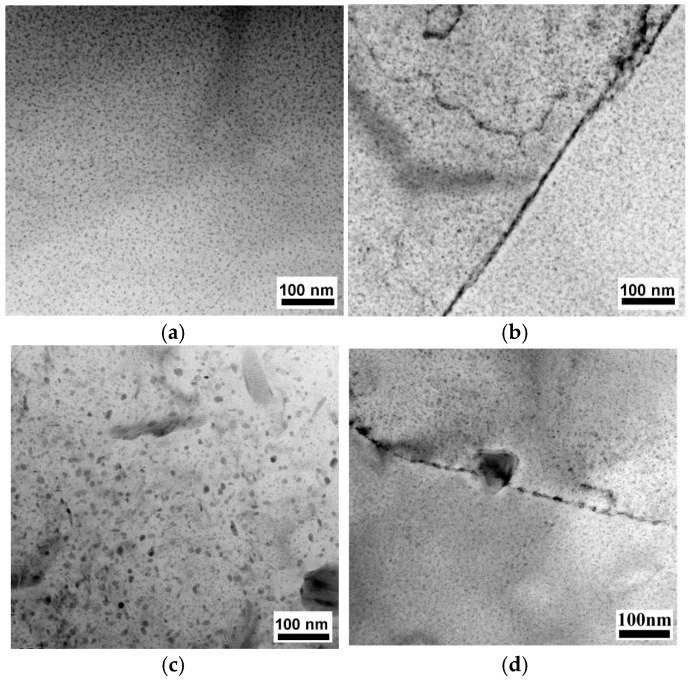
Photographs of intracrystalline and grain boundary precipitation phases of samples: (**a**,**b**) T6-processed and (**c**,**d**) MAF and aging.

**Figure 10:**
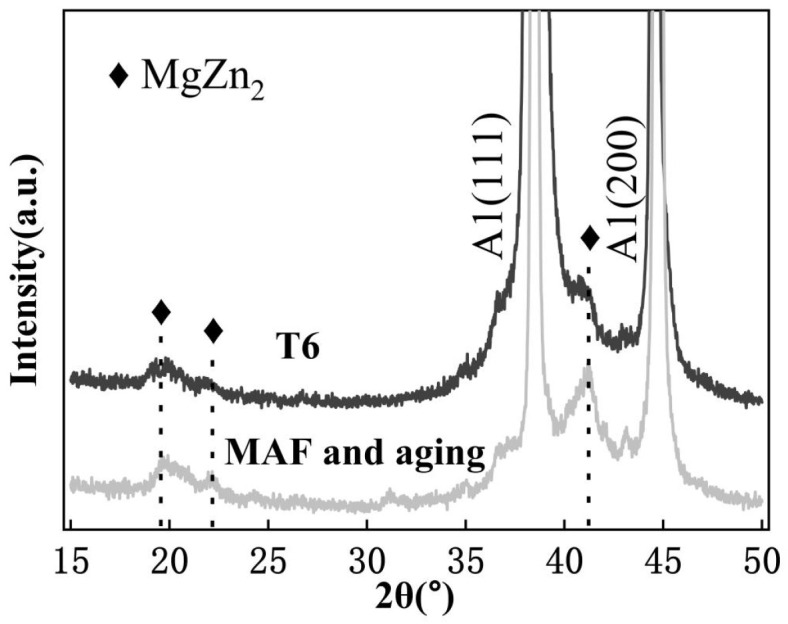
XRD patterns of the samples.

Figure 8 presents a TEM image of the samples. The microstructure of the MAF and aging sample with high dislocation densities, dislocation tangles, and some distinguishable irregularly shaped grains with a size ranging from a few microns is illustrated in Figure 8a. The corresponding diffraction pattern of the selected region suggests small misorientations among the fine grains, as shown in Figure 8b. Figure 9 illustrates the precipitates of the samples. The precipitates are homogeneously distributed in grains, as presented in Figure 9a,c. In the T6-processed sample, the grain boundary precipitates (GBPs) are smaller and continuous in distribution, as shown in Figure 9b. However, the larger and discontinuous distribution of GBPs is correlated with the MAF and aging sample, as exhibited in Figure 9d. Figure 10 presents X-ray patterns of the samples, which suggest that precipitates consist of MgZn2.

On the one hand, there are two reasons for the higher strength of the 7A52 aluminum alloy after MAF and aging. Firstly, during MAF, the sample is subjected to multidirectional severe strain, which causes dislocation multiplication and grain refinement [22]. Therefore, the increased strength of MAF processing is the comprehensive effect of dislocation strengthening and grain reduction strengthening. Secondly, the strengthening mechanism is due to precipitation strengthening after aging. On the other hand, there are several reasons for markedly improving the elongation of the MAF sample after aging. Firstly, aging causes recovery and grain growth, which leads to higher elongation. Secondly, the forging stresses of the MAF sample are reduced, which improves the elongation. Finally, it can be found that the mass of the precipitates is distributed within the grains in the MAF and aging sample, as presented in Figure 8b. These precipitates distributed within the grains are of benefit to the dislocation accumulation, which results in a high work hardening rate [23,24]. In tensile stress, a higher level of work hardening is essential for excellent elongation, as it contributes to the occurrence of local deformation (necking) delay [25].

Because aging enhances the diffusion of the kinetic energy of precipitates, the solute can be precipitated in the supersaturated solid solution. During the precipitation process, the precipitate depends on the fluctuation of the alloying element’s concentration and is considered to be homogeneous nucleation [26]. Homogeneous nucleation requires thermal activation above the high-energy barrier. Dislocation density is increased due to MAF processing. High elastic deformation energy is stored near the dislocations. Due to thermodynamic instability, the driving force of the phase transition is greatly increased, and the solute elements tend to aggregate near dislocations. The dislocations created by severe strain provide more heterogeneous nucleation sites for the precipitate formation [27,28]. The nucleation process is heterogeneous nucleation. The nucleation energy barrier is decreased, which results in the precipitation being accelerated, and the dispersion of precipitates is increased to form within the grains. Furthermore, MAF processing introduces a lot of new dislocations. Besides some dislocations exhibited in the grain, there are also some dislocations clustered around the grain boundaries. Apart from grain boundaries, dislocations also provide more heterogeneous nucleation sites for the nucleation of precipitates. Besides bulk diffusion, diffusion may also occur in the form of short-circuit diffusion. Short-circuit diffusion takes dislocation, grain boundary, and surface as diffusion paths. Short-circuit diffusion is also very important because its diffusion rate is much faster than that of bulk diffusion. When the dislocation density is higher, the formation rate of precipitates is accelerated, and the size of precipitates is increased. Therefore, the precipitates are large in size and discontinuously distributed at the grain boundary in the sample after MAF and aging processing, and are similar to those in the literature [29,30,31,32]. Moreover, the corrosion resistance of the AA7075 alloy may have been increased because of grain refinement, and the size of the precipitated-phase-free zone (PFZ) is reduced by adding TiB_2_ and TiC nanoparticles [33].

The IGC of aluminum alloys is mainly electrochemical corrosion, which can be attributed to the anode dissolution of GBPs or PFZ. The potential of the precipitate is different from that of the PFZ and matrix. Usually, the grain is the cathode, defects, impurities, and alloying elements, which are aggregated around the grain boundaries, resulting in a more reactive and negative electrode potential than that within the grains and becoming an anode; then, the micro-corrosion cell is formed, and IGC occurs [34]. The fine and continuous precipitates on the grain boundaries in the T6-treated sample act as anodic channels of corrosion and accelerate the IGC. As the GBPs are discontinuously distributed in the MAF and aging sample, it is hard to form the channels of anodic dissolution along the grain boundary, thus delaying the occurrence of corrosion along the grain boundary and improving the IGC resistance. In addition, a larger area fraction of GBPs is equivalent to an increase in the ratio of the anode relative to the cathode during the electrochemical reaction, which results in the anode current and dissolution rate of the GBPs decreasing [35], so the IGC resistance can also be increased.

EXCO is a special form of IGC. The causes of EXCO are the same as those of IGC, which also result from the formation of anode networks on the grain boundaries [35,36]. It is reported that the size and distribution of GBPs also have a very important influence on the EXCO [36]. In general, the sample shows higher IGC resistance, and it also has higher EXCO resistance. In this study, the testing results of EXCO are in accordance with those of IGC.

## 4. Conclusions

In this work, the hardness, tensile property, microstructures, and corrosion resistance of the 7A52 aluminum alloy are investigated after MAF and aging. The following main conclusions can be obtained from the present study:

(1) After MAF, the strength and elongation reached 532 MPa and 10.6%. The strength greatly increased and the elongation sharply decreased in the alloy. The strength was improved because of the dislocation multiplication and the grain size reduction.
(2) After MAF and aging, the strength and elongation reached 561 MPa and 12.3%. The strength was enhanced further due to precipitation strengthening. The elongation was improved by reducing the forging stresses. During aging, recovery and grain growth occur, which also results in higher elongation. Moreover, the precipitates were dispersed within the UFG, which increased the dislocation accumulation and work hardening. Therefore, the elongation could be enhanced as well.
(3) The UFG structure 7A52 aluminum alloy produced by MAF and aging with excellent corrosion resistance was mainly due to GBPs’ coarse and discontinuous distribution.

## Figures and Tables

**Table 1:** Chemical composition of 7A52 aluminum alloy (wt/%).

Zn	Mg	Mn	Cr	Cu	Fe	Zr	Ti	Si	Ni	Al
4.63	2.46	0.24	0.17	0.12	0.12	0.098	0.069	0.042	0.004	Bal

**Table 2:** Electrochemical polarization parameters of samples in 3.5% NaCl solution.

Samples	E_corr_ (V)	I_corr_ (A/cm^2^)	R_corr_ (Ω cm^2^)
T6	−0.896	6.508 × 10^−7^	15,807.1
MAF + aging	−0.886	5.714 × 10^−7^	22,633.2

## Data Availability

The original contributions presented in this study are included in the article. Further inquiries can be directed to the corresponding author.
